# Inhibition of histone deacetylases induces formation of multipolar spindles and subsequent p53-dependent apoptosis in nasopharyngeal carcinoma cells

**DOI:** 10.18632/oncotarget.9922

**Published:** 2016-06-08

**Authors:** Min Yan, Yuan-min Qian, Cai-feng Yue, Zi-feng Wang, Bi-cheng Wang, Wei Zhang, Fei-meng Zheng, Quentin Liu

**Affiliations:** ^1^ Sun Yat-sen University Cancer Center, State Key Laboratory of Oncology in South China, Collaborative Innovation Center for Cancer Medicine, Guangzhou, Institute of Cancer Stem Cell, Dalian, China; ^2^ Institute of Human Virology, Zhongshan School of Medicine, Sun Yat-sen University, Guangzhou, China; ^3^ Department of Laboratory Medicine, the First Affiliated Hospital, Sun Yat-sen University, Guangzhou, China; ^4^ Department of Medical Oncology, The First Affiliated Hospital, Sun Yat-sen University, Guangzhou, China

**Keywords:** histone deacetylases, p53, spindle, apoptosis, Nutlin-3

## Abstract

Histone deacetylases (HDACs) play crucial roles in the initiation and progression of cancer, offering a promising target for cancer therapy. HDACs inhibitor MGCD0103 (MGCD) exhibits effective anti-tumor activity by blocking proliferation and inducing cell death in malignant cells. However, the molecular mechanisms of HDACs inhibition induces cell death have not been well elucidated. In this study, we showed that MGCD effectively restored histone acetylation, suppressed cell growth and induced apoptosis in two-dimensional (2D) and three-dimensional (3D) cultured CNE1 and CNE2 nasopharyngeal carcinoma (NPC) cells. Importantly, MGCD arrested cell cycle at mitosis (M) phase with formation of multipolar spindles, which was associated with activated p53-mediated postmitotic checkpoint pathway to induce apoptotic cell death. Moreover, MGCD-induced apoptosis was decreased by inhibition of p53 using short interfering RNA (siRNA), suggesting that p53 was required for MGCD-induced cell apoptosis. Consistently, MGCD in combination with Nutlin-3, a MDM2 inhibitor showed synergistic effect on inducing apoptosis in 2D and 3D cultured CNE2 cells. Collectively, our data revealed that MGCD induced p53-dependent cell apoptosis following formation of multipolar spindles in NPC cells, suggesting the therapeutic potential of combinations of HDACs and MDM2 inhibitors for NPC treatment.

## INTRODUCTION

Histone deacetylases (HDACs) induce hypo-acetylated chromatin and transcriptional repression by removing the acetyl groups from the acetylated lysine in histones which results in gene silencing [1]. Recent evidence showed that dysregulation of HDACs and aberrant chromatin de-acetylation were tightly associated with tumorigenesis [2]. Aberrant recruitment of HDACs to promoters by oncogenic DNA-binding fusion proteins, or altered expression of individual HDACs in primary tumor samples had been reported in various cancers [3–4], indicating that HDACs might be attractive targets for therapeutic intervention. Consequently, HDACs inhibitors (HDACis) had been developed as promising target molecules for cancer therapy. Among these, MGCD0103, which inhibited HDAC1, 2, 3 and 11 selectivity showed appealing preclinical evidence of anti-cancer activity both *in vitro* and *in vivo* [5, 6]. Recently phase 1 and phase 2 studies of MGCD0103 had been completed in patients with malignancies and a well-tolerated safety profile had been demonstrated in these clinical trials [7–10].

Numerous studies showed that HDACis induced cell cycle arrest at G1/S through transcriptional activation of genes such as p21 and other cell cycle-regulated genes in a p53-independent manner [11, 12]. Emerging evidence indicated that HDACis could also induce G2/M cell cycle arrest in some human cancer cell lines [13, 14]. HDACis arrested cell cycle at G2/M phase was associated with disruption of pericentric heterochromatin and defects in spindle formation. Cells overrode mitotic spindle assembly checkpoint that leading to chromosomal instability [15–18]. Interestingly, p53 not only has an essential role in the G1 checkpoint in response to DNA-damaging agents such as radiation [19, 20], but also can be activated when damage occurs to the mitotic spindle. Indeed, microtubule disruption and spindle damage induce prolonged arrest of cellular mitosis, cause de-condensation of chromosomes and entry into ‘pseudo G1′ phase at the tetraploid DNA content. Subsequently, p53 is induced/activated via BubR1-mediated phosphorylation in these cells that eventually succumb to apoptotic cell death, which is mediated by p21^cip1/waf1^ in a similar way to its action in normal G1 phase to prevent replication of damaged DNA [21, 22]. Consistently, p53-deficient mouse embryo fibroblasts form multiploidy cells after spindle inhibitors treatment [23]. Thus, p53 functions as an essential postmitotic checkpoint following spindle disruption.

Interestingly, HDACs inhibited the function of p53 through reducing p53-DNA binding activity and specially down-regulating p53-dependent gene activation [24, 25]. Several HDACis such as butyrate and Valproic acid (VPA) were shown to restore p53 pathway without affecting its protein expression by nuclear re-localization and hyper-acetylation on lysine residues 373 and 382, which was thought to stabilize p53 in its active conformation [26]. On the other hand, SAHA was reported to exert anti-tumor effects by inducing polyploidy more markedly in p53−/− and p21−/− cells than in wild-type colon cancer cells [16]. These findings suggested that the anti-cancer activities of HDACis were tightly associated with p53 function/expression. However, the molecular mechanisms of the MGCD on the regulation of cell apoptosis through the spindle disruption-activated p53 pathways remained to be elucidated.

Moreover, HDACis have been shown to function synergistically with a host of structurally and functionally diverse anti-cancer agents both *in vitro* and *in vivo* experimental models and in the clinic [11]. For example, combination treatment using HDACis and retinoids was effective for the treatment of APL cells that were intrinsic and acquired resistant to retinoid acid alone [27]. The mutation or dysfunction of tumor suppressor p53 had been implicated as an initiating tumorigenic event. *In vivo*, p53 was tightly controlled by MDM2, which bound p53 protein with high affinity and negatively modulated its transcriptional activity and stability [28–30]. Thus, it seemed to be a reasonable molecular rationale for using HDACis in combination with Nutlin-3, a small molecule antagonist of MDM2, which effectively activated p53 pathway [31]. However, the potential anti-tumor efficacy of MGCD following addition Nutlin-3 has not been demonstrated.

In this study, we first showed that MGCD induced histone acetylation in both dose- and time-dependent manners in NPC cells, suggesting an efficacy of HDACs inhibition. In addition, MGCD was found to suppress proliferation and induce apoptosis in both 2D and 3D cultured NPC cells, indicating a potential molecular target for more selective therapeutic treatment in NPC. Importantly, we showed that MGCD induced multipolar spindles and arrested cell cycle at mitosis (M) phase, subsequently increased the expressions of p53 and its target gene p21 to activate Bcl-2 family-mediated apoptotic pathway. Inhibition of p53 by short interfering RNA (siRNA) resulted in reduction in cell apoptosis following treatment with MGCD, indicating that p53 was involved in MGCD-induced cell apoptosis in NPC cells. Furthermore, potent synergistic tumor cell apoptosis was observed using MGCD in combination with Nutlin-3 in 2D and 3D cultured NPC cells, suggesting a promising strategy for targeting therapeutics in cancers. Taken together, we reported that MGCD induced p53-dependent apoptosis following formation of multipolar spindles in NPC cells, providing a novel molecular basis of HDACis in cancer therapy.

## RESULTS

### MGCD suppresses proliferation in 2D and 3D cultured NPC cells

To verify that MGCD *inhibitory* activities of HDACs, we first examined the effect of MGCD on the acetylation of histones by western blot analysis. As shown in Supplementary Figure 1A, incubation of exponentially growing CNE2 cells with MGCD for 24 and 48 h led to both dose- and time-dependent increase in the level of Ac-Histone H3. Similarly, MGCD also induced histone H3 acetylation in other NPC cell lines including CNE1, SUNE1 and HK1 (Supplementary Figure 1B and 1C), demonstrating that MGCD effectively inhibited HDACs activities in NPC cells.

**Figure 1:**
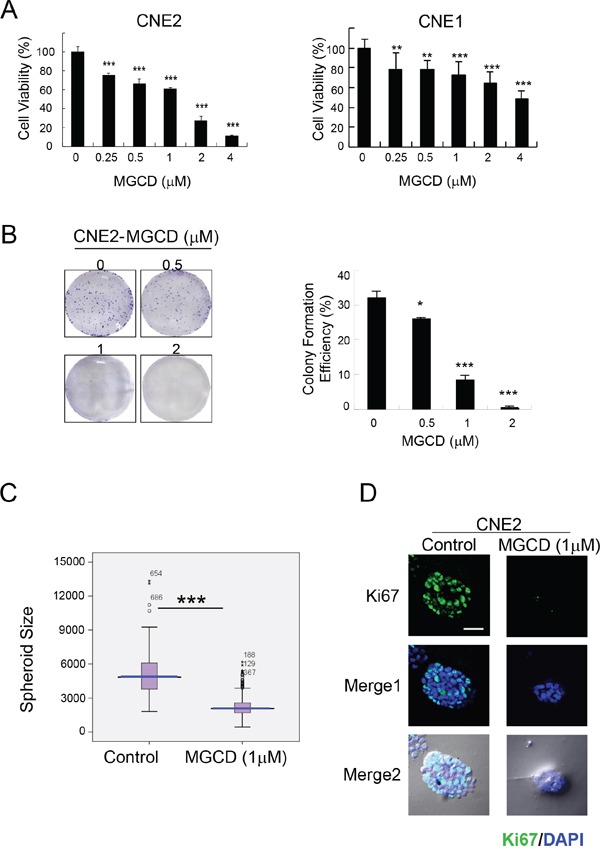
MGCD suppresses proliferation in 2D and 3D cultured NPC cells **A.** CNE2 and CNE1 cells were cultured in indicated doses of MGCD and assayed at 72 h using the MTT assay. Data were the mean ± SD of three independent experiments, **p<0.01; ***p<0.001. **B.** The colony formation ability of CNE2 cells decreased in MGCD-treated cells (*p<0.05; ***p<0.001), left panels were the representative images of wells with colonies. For colony formation assay, CNE2 cells were cultured with different concentration of MGCD for 14 days. **C.** Distribution of spheroid size (circumferential area) of 3D cultured spheroid structures grown in DMSO (control) or 1 μM MGCD. Area of each spheroid was measured using Olympus Image-Pro Plus 6.0 software and plotted as box plots. Blue line, median value; spread, 1.5 times the interquartile range; circles, outliers. Each condition represents ~200 spheroids structures from three independent experiments, ***p<0.001. **D.** Confocal immunofluorescence images of 3D cultured CNE2 cells grown in DMSO (control) or 1 μM MGCD 6 days were stained with proliferative marker Ki67 (green). The nuclei were stained with DAPI (blue). Scale bar, 50 μm. Results shown are representative of three independent experiments.

Next, we examined the effect of MGCD on the growth and viability of 2D and 3D cultured NPC cells. MGCD inhibited cell growth in 2D cultured CNE2 and CNE1 cells in a dose-dependent manner as assessed by MTT assay (Figure 1A). Colony formation assay showed that MGCD at concentration of 1 μM potently inhibited >50% clonogenic growth of CNE2 cells (Figure 1B). 3D spheroid structures formed by MGCD-treated CNE2 cells were smaller than those formed by control cells (Figure 1C). We then detected the expression level of the proliferation marker Ki67 during 3D morphogenesis. Cells in spheroid incubated with 1 μM MGCD for 6 days had low proliferation rates shown as Ki67 negative (Figure 1D). These data indicated that MGCD significantly suppressed proliferation of NPC cells.

### MGCD induces apoptosis in 2D and 3D cultured NPC cells

**Figure 2:**
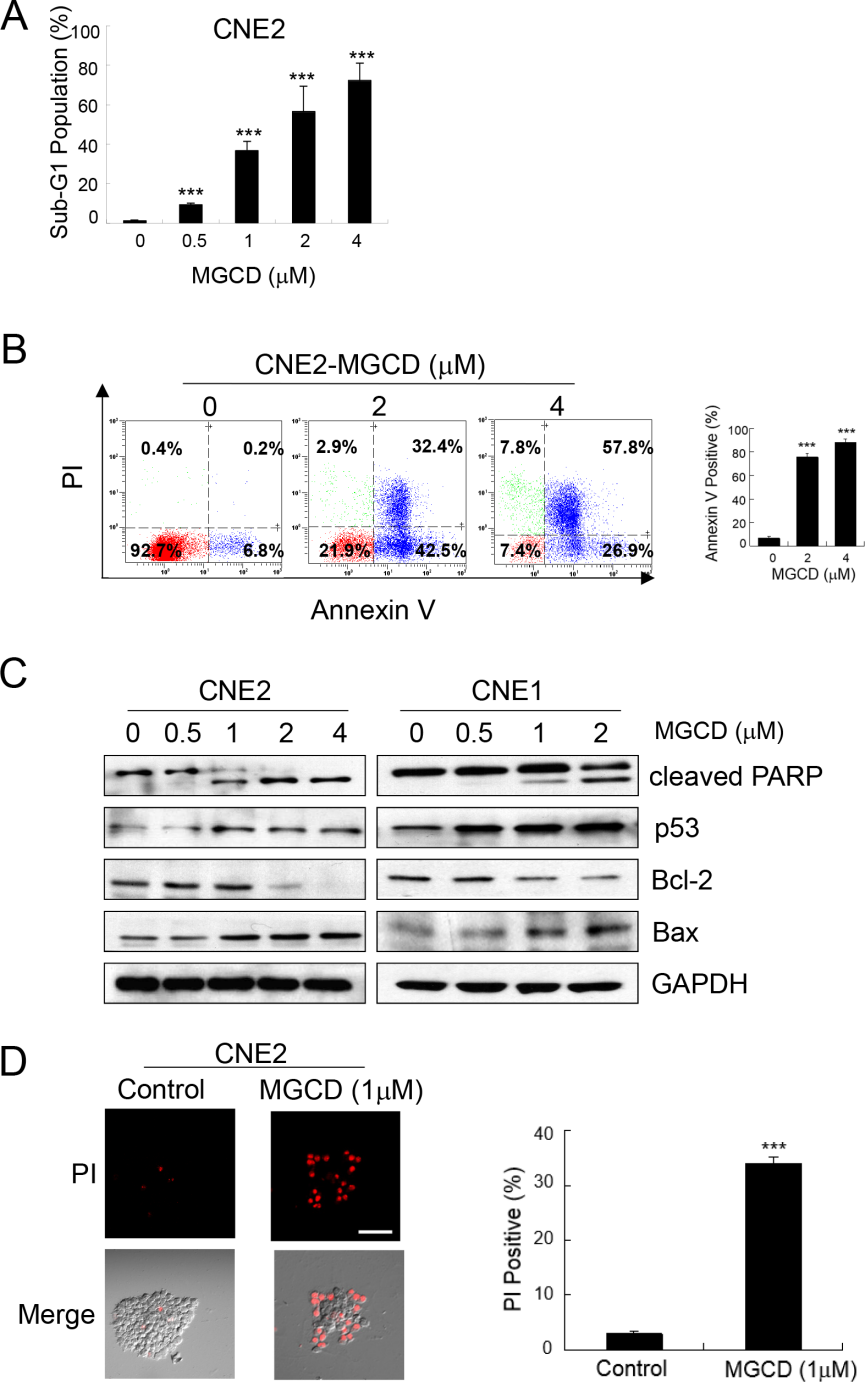
MGCD induces apoptosis in 2D and 3D cultured NPC cells CNE2 or CNE1 cells were treated with indicated MGCD or DMSO (control) for 48 h. **A.** MGCD-induced apoptosis in CNE2 cells by sub-G1 analysis. Exposure to MGCD caused significant increases in sub-G1 DNA content of CNE2 cells at increasing concentrations. Histogram represented quantitative analysis of the percentage of sub-G1 populations from three independent experiments. Bars, SD, ***p<0.001. **B.** Flow cytometric analysis of apoptosis in CNE2 cells incubated with MGCD were measured by Annexin V/PI analysis. Representative scatter plots showed the distribution of Annexin V and PI staining for control and MGCD-treated CNE2 cells (left panel). Quantitative analysis of the percentage of Annexin V positive cells from three independent experiments. Bars, SD, ***p<0.001. **C.** Western blot analysis of CNE2 and CNE1 cells were treated with indicated MGCD concentration for 48 h for indicated antibodies. GAPDH served as loading control. **D.** 3D cultured CNE2 cells were incubated with DMSO (control) or 1 μM MGCD from day 4 to day 6, the spheroid structures then subjected to PI staining (red), scale bar, 50 μm. Histogram was quantitative analysis of the percentage of PI positive cells from three independent experiments. Error bars indicated the SD, ***p <0.001.

To further test the effect of MGCD, we studied the consequences of MGCD 48 h treatment on NPC cells. The ability of MGCD to induce apoptosis was assessed by flow cytometry analysis. Treatment of MGCD (0.5 − 4 μM) for 48 h induced sub-G1 fractions in a dose-dependent manner (Figure 2A). In addition, significant (*p*<0.001) increase of Annexin V positive cells were seen after 48 h exposure to MGCD at 2 and 4 μM in CNE2 cells (Figure 2B). Western blot analysis showed that incubation of MGCD for 48 h led to increase cleaved caspase-3 in a dose-dependent manner in CNE2 and CNE1 cells (Supplementary Figure 2). Elevated level of cleaved PARP, a major target for caspases was detected in MGCD treatment (Figure 2C). Moreover, in a dose-dependent manner, the expression of p53 and pro-apoptotic factor Bax were increased and the level of anti-apoptotic protein Bcl-2 was decreased in MGCD-treated CNE2 cells. Similar results were obtained in CNE1 cells (Figure 2C). Furthermore, MGCD induced cell death in 3D cultured CNE2 cells as shown as PI positive population (Figure 2D). These observations suggested that MGCD induced apoptotic cell death via impairing the balance of Bcl-2 family members in caspase activation pathways.

### MGCD blocks cell cycle at M phase by formation of multipolar spindles in CNE2 cells

**Figure 3:**
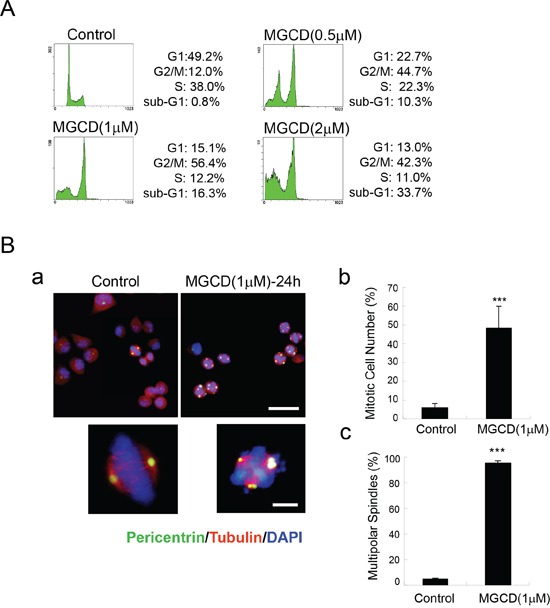
MGCD induces multipolar spindles and cell cycle M phase arrest in CNE2 cells **A.** Cell cycle M phase arrest induced by MGCD in CNE2 cells incubated with DMSO (control) or various doses of MGCD for 24 h. Cell cycle distribution was analyzed by flow cytometry. One representative of three independent experiments was shown. **B.** Confocal immunofluorescence images of CNE2 cells incubated with DMSO (control) or 1 μM MGCD for 24 h were stained with pericentrin (green), α-tubulin (red), DAPI (blue) was used to visualize the nuclei (a; top panel, Scale bar, 50μm; bottom panel, Scale bar, 10 μm). Quantification showed the percentage of mitotic cells (b) and the abnormal spindles assessed as multipolarity (c) of three independent experiments. For (b) percentage of mitotic cells was calculated by counting the mitotic cells per one hundred cells. For (c) percentage of multipolar spindles was calculated by counting the number of cells with abnormal spindle per one hundred mitotic cells. Error bars indicated the SD, ***p<0.001.

To explore the mechanism of anti-proliferative effects of MGCD, we further performed cell cycle analysis. Flow cytometry assay results showed that incubation of MGCD for 24 h resulted in cell cycle G2/M phase arrest in CNE2 cells (Figure 3A). Accordingly, 24 h exposure of CNE2 cells to MGCD was associated with increased amounts of Cyclin B1 and decreased levels of p-cdc2 (Tyr15), indicating that the MGCD-treated CNE2 cells were blocked at G2/M phase (Supplementary Figure 3). Additionally, immunofluorescence staining revealed that MGCD induced formation of multipolar spindles (Figure 3B-a). The mitotic index in MGCD treated cells was significantly higher than control cells (Figure 3B-b). Moreover, most of the MGCD-treated mitotic CNE2 cells displayed multipolar spindles (Figure 3B-c). Similar results were obtained in CNE1 cells (data not shown). These data indicated that MGCD could induce defects in mitotic spindle formation.

### MGCD induces apoptosis in CNE2 cells through p53-mediated postmitotic checkpoint pathway

**Figure 4:**
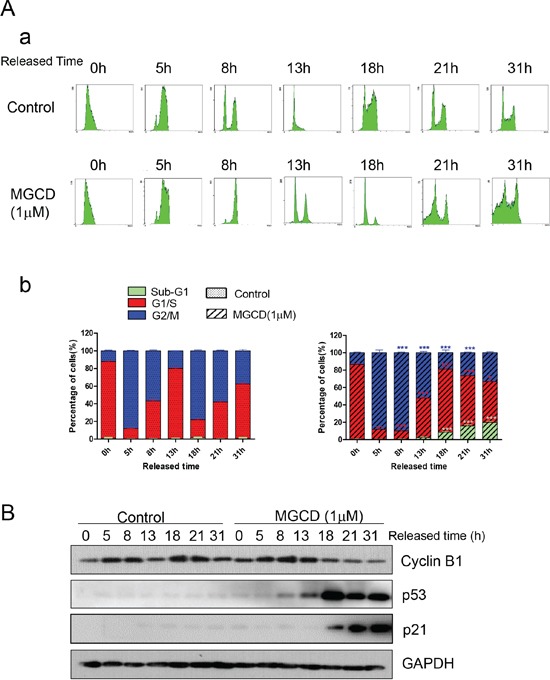
Time-lapse detection of CNE2 cells in the presence or absence of MGCD CNE2 cells were synchronized by use of double thymidine for G1/S blocking, then released in control medium (DMSO) or medium containing 1 μM MGCD and incubated for various times, **A.** cells were stained with propidium iodide (PI), then analyzed by flow cytometry. Shown were representative histograms (a) and the number of cells in the sub-G1, G1/S, or G2/M-phase is given as percentages of the total cell population (b). Data were mean ± SD of three independent experiments, *p<0.05; ***p<0.001. **B.** Cell lysates were prepared and subjected to western blot analysis for Cyclin B1, p53 and p21. GAPDH was used as loading control.

**Figure 5:**
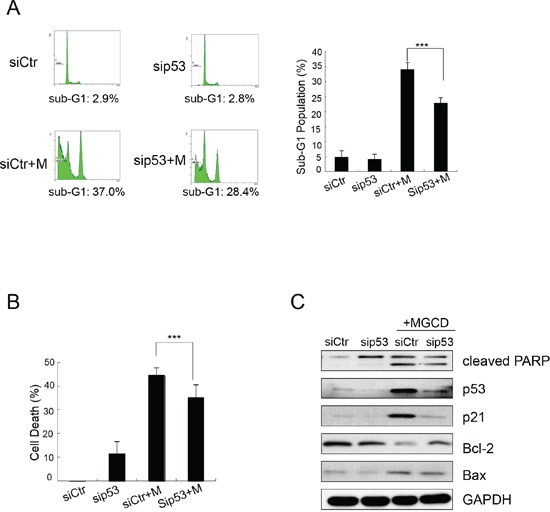
MGCD induces apoptosis by up-regulating p53 in CNE2 CNE2 cells transfected with siRNA targeted against p53 (sip53) or control siRNA (siCtr) were treated with 2 μM MGCD for 24 h. **A.** Apoptosis of MGCD-induced sip53 and siCtr CNE2 cells was assessed by sub-G1 analysis. Shown were representative histograms of three independent experiments (left panel) and quantification of three independent experiments (right panel, ***p <0.001). **B.** Cell death was determined by trypan blue assay. Columns represented the mean of three independent experiments; bars, SD; ***p <0.001. **C.** Western blot analysis checked the expression level of cleaved PARP, p53, p21, Bcl-2 and Bax. GAPDH was used as loading control.

As shown in Figure 3, MGCD induced multipolar spindles, subsequently blocked cell cycle at M phase, which was closely associated with chromosome instabilities [23]. Recently, p53 was reported to function as a postmitotic spindle checkpoint, playing a crucial role in maintaining chromosome diploidy [21, 23]. To gain insight into the mechanisms of MGCD induced apoptosis, we performed western blot assay to analyze the expression of p53 and p21. Inhibition of HDACs by MGCD enhanced the cellular levels of p53 and p21, a major transcription target of activated p53 [32] in a dose-dependent manner (Supplementary Figure 4). Moreover, by performing synchronization assay, we found that both Control and MGCD-treated cells entered M phase at 5 h after released, and the expression of Cyclin B1 fluctuated according to cell cycle (Figure 4A and 4B). At 8 h after released, Control cells have begun to reenter G1/S phase. However, MGCD prolonged cellular mitosis, consistent with their ability to induce formation of multipolar spindles. MGCD induced expression of p53 and p21 after cells exit mitotic arrest (Figure 4B), which prevented cells reentering another cell cycle. And subsequently, it induced cell apoptosis shown as increased sub-G1 population (Figure 4A). These data suggested that MGCD activated a p53-mediated postmitotic checkpoint pathway following spindle disruption. To determine whether p53 was required for MGCD-induced-apoptosis in NPC cells, we knocked down p53 by siRNA in CNE2 cells. As shown in Figure 5A, MGCD-induced apoptosis was reduced in p53 depleted CNE2 cells than control cells as measured by sub-G1 population. Similar results were observed in viability cell count assay (Figure 5B). Meanwhile, western blot analysis showed that MGCD treatment resulted in increased Bax expression and decreased Bcl-2 level, which were reversed in p53 depleted CNE2 cells (Figure 5C). These results suggested that p53 was involved in MGCD-induced apoptosis in CNE2 cells.

### Nutlin-3 synergizes MGCD-induced-apoptosis in 2D and 3D cultured CNE2 cells

**Figure 6:**
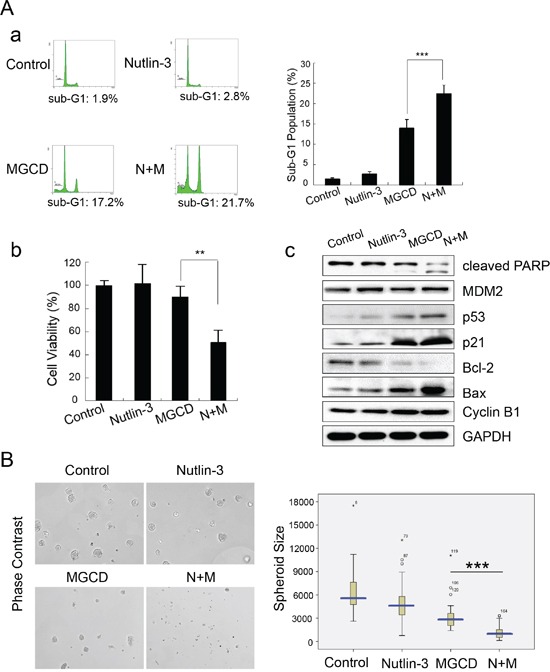
Nutlin-3 synergizes MGCD-induced-apoptosis in 2D and 3D cultured CNE2 cells **A.** CNE2 cells treated with MGCD (1 μM), Nutlin-3 (10 μM) alone or in combination for 24 h. (a) Apoptosis was assessed by sub-G1 analysis. Shown were representative histograms of three independent experiments (left panel) and quantification of three independent experiments (right panel, ***p <0.001). (b) Cell viability was determined by trypan blue assay. Columns represented the mean of three independent experiments; bars, SD; ***p <0.001. (c) Western blot analysis checked the expression level of cleaved PARP, p53, p21, MDM2, Bcl-2, Bax, and Cyclin B1. GAPDH was used as loading control. **B.** CNE2 cells were subjected to 3D culture assay, and treated with DMSO (control), MGCD (1 μM), Nutlin-3 (10 μM) alone or in combination from day 4 to day 6. Representative phase-contrast micrographs of spheres of day 6 (left panel). Distribution of 3D cultured spheroid size (circumferential area, right panel). Data were the mean ± SD of three independent experiments, ***p<0.001.

Next, we introduced Nutlin-3, a small-molecule MDM2 inhibitor, which could inhibit p53-MDM2 interaction and lead to p53 stabilization [31]. Interestingly, MGCD and Nutlin-3 in combination showed synergetic effect on reducing cell viabilities and inducing apoptosis compared with MGCD or Nutlin-3 alone (Figure 6A-a). Accordingly, treatment of CNE2 cells with MGCD (1 μM) in the presence of Nutlin-3 (10 μM) for 24 h significantly decreased cell viability (Figure 6A-b), increased the cellular level of cleaved PARP, Bax and p21 as well as decreased Bcl-2 expression (Figure 6A-c) as measured by western blot assay. We performed 3D cultured assay and obtained similar results (Figure 6B). In the presence of MGCD combined with Nutlin-3, CNE2 cells could hardly form 3D spheroid structures. These observations indicated a therapeutic strategy of combinations of HDACis and MDM2 inhibitors for NPC treatment.

## DISCUSSION

HDACs are promising targets for therapeutic interventions intended to reverse aberrant epigenetic states which is closely associated with tumorigenesis [33]. HDACis have been reported to effectively inhibit proliferation, induce differentiation [27, 34], suppress tumor angiogenesis, metastasis and invasion [35–37]. However, the potential role of HDACis in NPC treatment and the molecular mechanisms of MGCD induced mitotic defect and apoptosis has not been evaluated. In the present study, we found that selective HDACi MGCD enhanced acetylation of histone H3 in NPC cells. In both dose- and time-dependent manners, MGCD inhibited cell growth and induced apoptotic cell death in 2D and 3D cultured NPC cells. Most importantly, MGCD induced spindle disruption and blocked cell cycle at M phase, which subsequently activated p53-mediated postmitotic checkpoint to induce cell apoptosis. Moreover, we showed that MGCD in combination with Nutlin-3, a MDM2 inhibitor, resulted in a synergetic effect on inducing apoptosis in NPC cells. Thus, these data revealed a novel mechanism that MGCD induced p53-dependent apoptosis in NPC cells following spindle disruption and defects in mitosis, indicating HDACs serve as a potential therapeutic target in NPC.

HDACis were first reported to induce cell-cycle arrest at G1/S boundary mediated by retinoblastoma protein (pRb) and p21 in a p53-independent manner [38]. The induction of DNA damage gene GADD45α/β [39] and many cell cycle regulatory genes such as the CDK inhibitors p21, p15INK4B, p19INK4D and p57 [2, 35] was involved in HDACis induced cell cycle disruption. Another study showed that HDACis activated TGFβ receptor signaling, which led to cell cycle arrest and apoptosis [40]. In this study, we showed that MGCD induced abnormal spindles as multipolar and defects in mitosis, led to cell cycle arrest at M phase, suggesting that MGCD exerted its anti-tumor activity by interfering with mitotic progression. These data were consistent with several recent studies reported that HDACs inhibition caused G2/M arrest and mitosis defect in different cancer cells [13, 41]. Recent research found that this effect of HDACs inhibition was associated with inducing pericentromeric histone hyperacetylation resulted in altered kinetochore assembly [15, 42] and inhibition of Aurora A/B kinases led to mitotic defect [43–45], which was independent of ongoing gene transcription, suggesting that the hyperacetylation of histone induced by HDACis directly interfered with mitotic progression. Interestingly, one study reported that cells with defective mitotic checkpoint were more susceptible to chromosomal instability by inhibiting HDACs activity [17]. Thus, these findings supported that HDACis exerted anti-tumor functions by directly targeting mitosis on histone acetylation levels. As cancer cells usually have a defect in cell cycle checkpoints, this might determine HDACis' advantage of tumor-selective effect.

Numerous studies have shown that HDACis exerted anti-tumor activities by inducing apoptosis in cancer cells. The molecular pathways of HDACis-mediated cell death through multiple mechanisms including activation of the intrinsic and extrinsic apoptosis pathways, accumulation of DNA damage or ROS, as well as induction of immunogenic cell death [2]. Considering that p53 plays key roles in initiating intrinsic apoptotic pathway and HDACs destabilizes p53 by deacetylation [24, 25], HDACis could augment p53 function of pro-apoptosis. In this study, we showed that MGCD decreased the expression of anti-apoptotic protein Bcl-2 and increased the level of pro-apoptotic protein Bax, induced activation of caspase-3 and PARP cleavage, supporting a crucial role the mitochondrial apoptotic pathway plays in MGCD mediated tumor cell death. In addition, we showed the expression of p53 and its transcription target p21 was increased dose-dependent in MGCD-treated cells. Furthermore, p53 was required for MGCD-induced apoptosis, consistently with several recent studies indicated that HDACis-mediated cell death was p53-dependent [26, 46]. A very interesting report found that HDACis could induce a p53-mediated pro-apoptotic response and a host of p53 target genes expression such as p21 and Bax by regulating the activity of both wild-type and mutant p53 [47]. These data indicated a strong case for the use of HDACis in tumors that bearing functionally inactive p53.

Both genetic and epigenetic alterations play critical roles in tumor initiation and progression. Recent studies indicated that HDACis were effectively against a defined subset of hematological tumors, however, there was less than convincing evidence in solid tumors [48, 49]. Combination therapy strategy had shown promise for the treatment of NPC patients [50]. Considering the range of molecular and biological responses mediated by HDACis and minimal toxicity to normal cells, the application of HDACis combined with other anti-cancer agents is proved to be the most useful application. Although HDACis have been shown to augment anti-cancer activities of a plethora of pharmacological and biological anticancer agents, the molecular mechanisms that underpin synergistic combination effects are largely ill-defined [11, 51, 52]. Thus, exploring the molecular rationale for combination using HDACis will provide useful information for future clinical studies. We demonstrated that MGCD in combination with Nutlin-3 induced synergistic apoptosis in CNE2 cells accompanying with increased-p53 expression, suggesting that the ability of Nutlin-3 to potentiate the anticancer activity of MGCD was intimately linked to prevent p53 degradation. These data were consistent with one recent report that HDACis Trichostatin A (TSA) and SAHA combined with proteasome inhibitors showed synergistic effect on HPV-positive cervical cancer cells, coinciding with elevated level of p53 *in vitro* and *in vivo* [53]. As deregulation of the p53 pathway is crucial for early tumorigenesis and may contribute to drug resistance following chemotherapy [54], our data may reinforce the existing anti-tumor therapy via bypassing such impairment.

Taken together, our results demonstrated that HDACi MGCD effectively suppressed proliferation, induced apoptosis in 2D and 3D cultured NPC cells. MGCD induced apoptosis in a p53-dependent manner resulting from formation of multipolar spindles and mitotic arrest. Combination of MGCD and Nutlin-3 showed synergistic effect of inducing apoptosis in NPC cells, suggesting a promising therapeutic strategy for targeted cancer therapy in the near future.

## MATERIALS AND METHODS

### Reagents and antibodies

MGCD was kindly provided by ROCHE R & D CENTER (CHINA) LTD. Nutlin-3 was purchased from Sigma. For western blot detection, we used Ac-Histone H3 (Upstate), Histone H3, Cleaved caspase-3, Bcl-2, Cyclin B1, Bax (Epitomics), p-cdc2 (Tyr15), cdc2, p53, p21 (Cell Signaling Technology), cleaved PARP (Santa Cruz), and GAPDH, tubulin (Proteintech Group) antibodies.

### Cell culture

CNE2, CNE1, SUNE1 and HK1 cell lines were obtained from Dr Chaonan Qian (Sun Yat-sen University, Guangzhou, China) as described previously [55]. The NPC cell lines CNE1, CNE2, SUNE1 and HK1 cells were maintained in RPMI 1640 (Invitrogen) supplemented with 10% fetal bovine serum (Hyclone). The cells were incubated at 37°C in a humidified chamber containing 5% CO_2_.

### Western blot

Cells were lysed in 1.25% SDS; 0.0125 NaPO_4_ (pH 7.2); 50 mmol/L NaF; 2 mmol/L EDTA; 1.25% NP40; 1 mmol/L sodium vanadate, and a pellet of complete protease inhibitor mixture [15] and centrifuged at 14,000 g for 10 min at 4°C to remove insoluble material, boiled (20 μg for each sample) for 5 minutes, separated by 10-12% SDS-PAGE, transferred to nitrocellulose membrane (Millipore), and immunoblotted with the indicated antibodies. Horseradish peroxidase-conjugated goat anti-mouse or goat anti-rabbit IgG (Pierce) were used as secondary antibodies. Proteins were visualized with a Super Signal West Pico chemiluminescence kit (Pierce).

### MTT assay

Cells were seeded at 5000 cells/well onto 96-well plates and incubated with different doses of MGCD. After 72 h, cells were subjected to MTT assay as described previously [56].

### Colony formation assay

CNE2 cells were mixed in RPMI 1640 media supplemented with 10% FBS and plated at 1000 cells/well onto 6-well plates. After 14 days, colonies were dyed with crystal violet (Sigma), photographed and counted.

### Immunofluorescence staining

Cultured cells were fixed with 2% formalin (Sigma) for 20 min at room temperature (RT), blocked with 1% BSA for 30 min, stained sequentially with primary antibodies against pericentrin (Abcam) and tubulin (Sigma) for 1 h at RT, and then incubated with secondary antibodies conjugated to Alexa-488 or −680 for 30 min. The nuclei were stained for 15 minutes with PBS containing 1 μg/ml of 4, 6-diamidino-2-phenylindole (DAPI, Sigma) before mounted with the anti-fade agent Prolong (Molecular Probes). Confocal analyses were performed with Olympus IX81 confocal microscopy systems.

### Flow cytometry assay

For cell cycle detection, cells were collected and fixed, and resuspended in cell cycle buffer (0.38 mM sodium citrate, 0.5 mg/mL RNase A, and 0.01 mg/mL PI). For apoptotic analysis, cells were collected, stained with Annexin V (Annexin V-FITC Apoptosis Detection Kit, EMD Biosciences, San Diego, CA) according to the manufacturer's protocol and subjected to FACS analysis.

### Synchronization

A double thymidine addition was used for G1/S-phase synchronization. Cells were incubated with thymidine at a final concentration of 2 mM for 12 h. The thymidine-containing medium then was replaced with normal culture medium, and the cells were grown for 16 h before adding 2 mM thymidine again. Cells were incubated for a further 12 h to synchronize the cells at the G1/S border. The arrest was subsequently released by growing the cells in thymidine-free medium.

### SiRNA

Cells were transfected with p53-siRNA (5′-CUACUUCCUGAAAACAACGdTdT-3′) or control scramble RNA (5′-UUCUCCGAACGUGUCACGUTT-3′) duplex (4 μM/2×10^6^ cells) by Lipofectamine 2000 (Invitrogen), and lysates prepared 24-48 h after transfection.

### Viability cell count assay

Cells after incubated with indicated conditional media were collected, stained with Trypan Blue and subjected to cell count. Data were calculated as the means of at least three independent experiments.

### 3D cultures and immunofluorescence assay

For 3D cultures assay, cells were cultured according to manufacturer's instructions. Briefly, 1,000 single cells per well were plated in an eight-well chamber slide (BD Biosciences) coated with Matrigel from BD Biosciences. Cells were grown in assay medium with DMSO or 1 μM of MGCD. Medium was changed every 4 days. For 3D immunofluorescence, the cell spheroids were fixed with 2% formalin (Sigma) for 20 min at RT, permeabilized with PBS containing 0.5% Triton X-100 for 10 min at 4°C, and blocked in blocking Buffer (130 mM NaCl, 7 mM Na_2_HPO_4_, 3.5 mM NaH_2_PO_4_, 7.7 mM NaN_3_, 0.1% BSA, 0.2% Triton X-100, 0.05% Tween-20, 10% goat serum) for 45-60 min at RT, and incubated with primary antibody against Ki67 (BD Biosciences) for 15-18 h at 4°C, then incubated with fluorescent conjugated secondary antibody for 40-50 min at RT. DAPI was used to stain nuclei. Spheroid structures were imaged and analyzed using Olympus IX81 confocal microscopy systems [55].

### Statistical analysis

Statistics were calculated by SPSS software (version 17.0). Statistical significance was analyzed by Student's t test and expressed as *p* value. For 3D cultures, the spheroid size analyses were performed using GraphPad Prism software and Mann-Whitney test.

## SUPPLEMENTARY FIGURES


