# Supplementary material for: Inhibition of histone deacetylases induces formation of multipolar spindles and subsequent p53-dependent apoptosis in nasopharyngeal carcinoma cells
Source: Oncotarget. 2016 Jun 8;7(28):44171–84. doi: 10.18632/oncotarget.9922 (PMC5190087; doi:10.18632/oncotarget.9922)
Supplement: Supplementary file 1 [file oncotarget-07-44171-s001.pdf]

Inhibition of histone deacetylases induces formation of multipolar spindles and subsequent p53-dependent apoptosis in nasopharyngeal carcinoma cells

SUPPLEMENTARY FIGURES

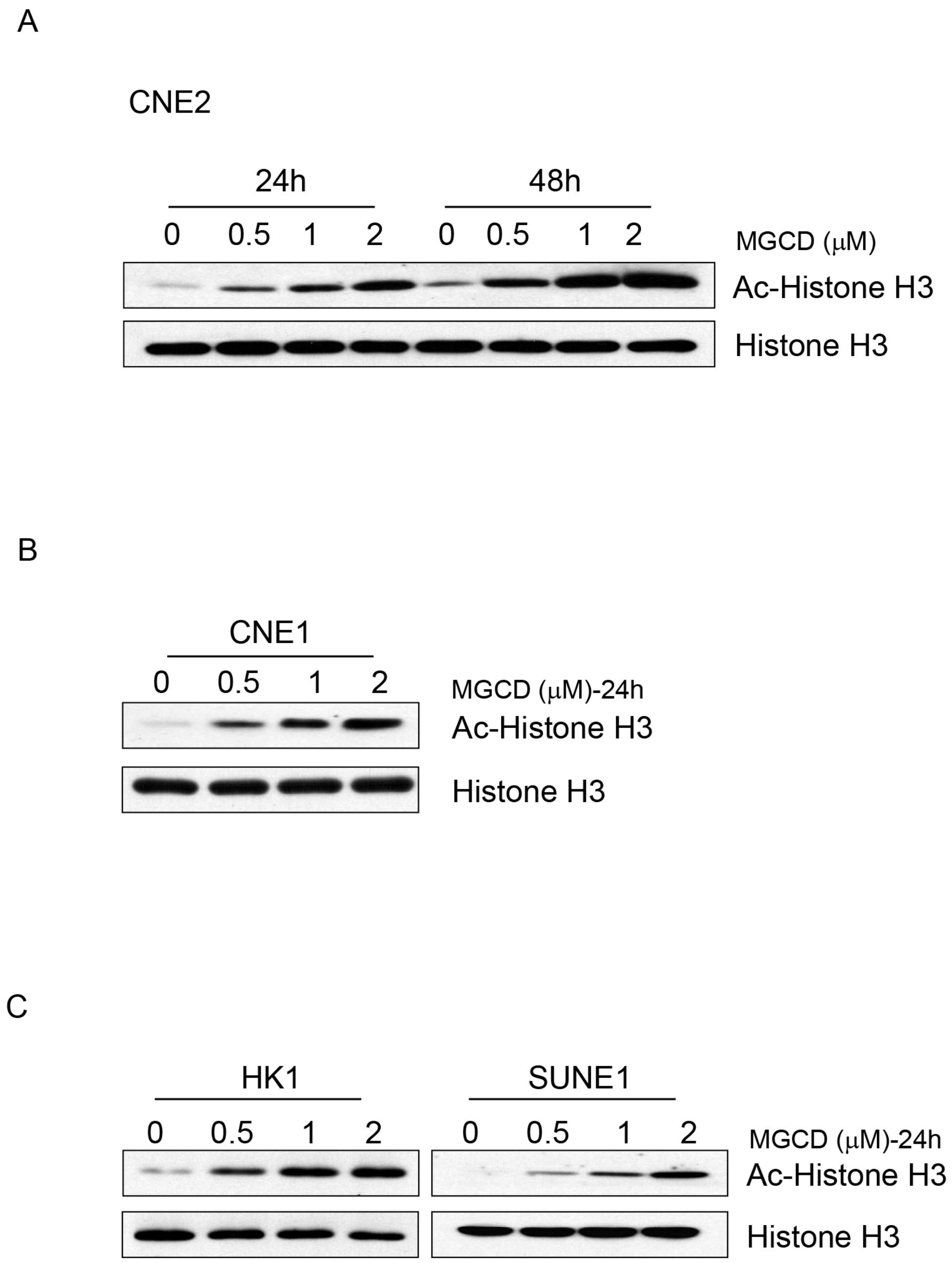

Supplementary Figure S1: MGCD induces histone acetylation in NPC cells. CNE2 (A), CNE1 (B), HK1 and SUNE1 cells (C) were incubated with DMSO (served as control) or indicated doses MGCD for 24 h or 48 h, and subjected to Western blot with Ac-Histone H3 antibody. Histone H3 was used as loading control.

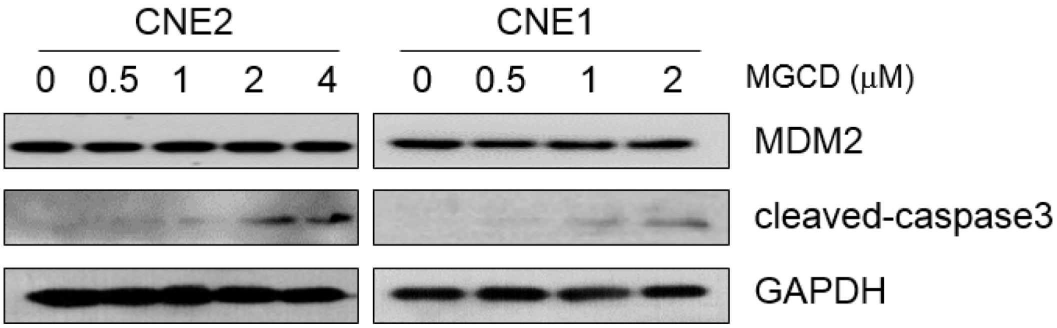

Supplementary Figure S2: MGCD induces cleaved-caspase3 and has no effect on MDM2 in NPC cells. CNE2 and CNE1 cells were incubated with indicated doses of MGCD for 48 h before harvesting. Cell lysates were analyzed by Western blot with cleaved-caspase3 and MDM2 antibodies. GAPDH was used as loading control.

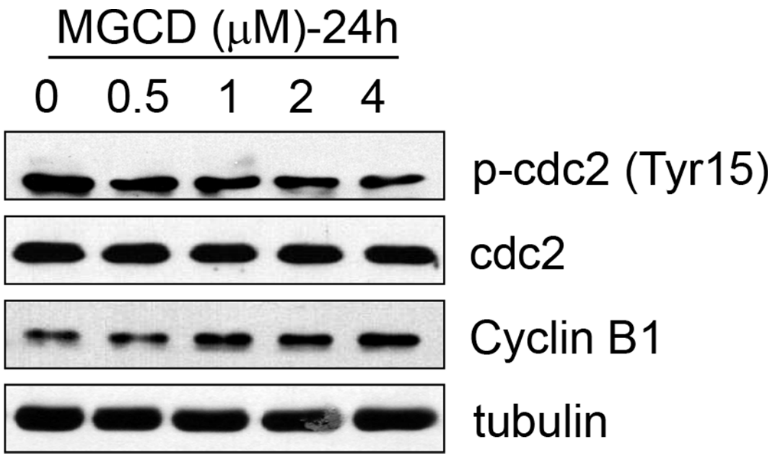

Supplementary Figure S3: MGCD decreases p-cdc2 and increases Cyclin B1 expression in CNE2 cells. CNE2 cells were treated with MGCD at indicated doses for 24 h and subjected for Western blot with p-cdc2 (Tyr15), cdc-2 and Cyclin B1 antibodies. Tubulin was used as loading control.

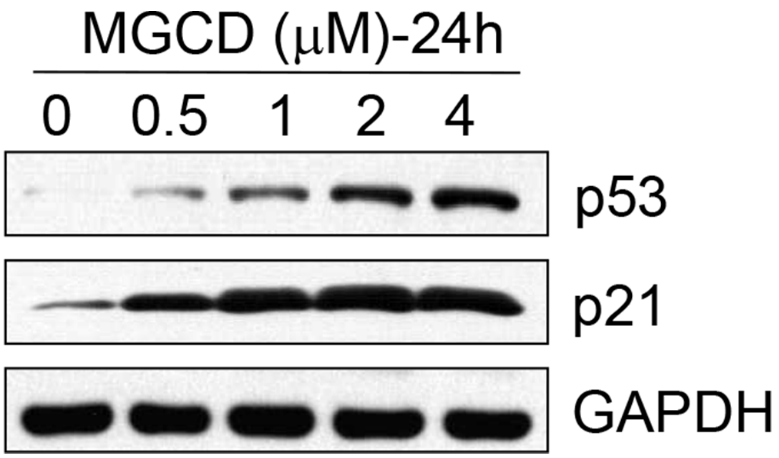

Supplementary Figure S4: MGCD increases p53 and p21 in CNE2 cells in a dose-dependent manner. CNE2 cells were incubated with MGCD at indicated doses for 24 h before harvesting. Cell lysates were subjected for Western blot assay with p53 and p21 antibodies. GAPDH was used as loading control.
